# “That’s a woman’s problem”: a qualitative analysis to understand male involvement in maternal and newborn health in Jigawa state, northern Nigeria

**DOI:** 10.1186/s12978-019-0808-4

**Published:** 2019-09-18

**Authors:** Vandana Sharma, Jessica Leight, Nadège Giroux, Fatima AbdulAziz, Martina Bjorkman Nyqvist

**Affiliations:** 1 Abdul Latif Jameel Poverty Action Lab, Massachusetts Institute of Technology, 400 Main Street E19-201, Cambridge, MA 02142 USA; 2 Present Address: Harvard T.H. Chan School of Public Health, 677 Huntington Avenue, Boston, MA 02115 USA; 3 Poverty, Health and Nutrition Division, International Food Policy Research Institute, Washington, DC USA; 4 Paris School of Economics, Paris, France; 5 Planned Parenthood Federation of Nigeria, Abuja, Nigeria; 6 Department of Economics, Stockholm School of Economics, Stockholm, Sweden

**Keywords:** Male involvement, Maternal mortality, Neonatal mortality, Maternal complications, Newborn complications, Recognition, Care-seeking, Jigawa, Nigeria, Sub-Saharan Africa

## Abstract

**Background:**

Maternal and newborn mortality continue to be major challenges in Nigeria. While greater participation of men in maternal and newborn health has been associated with positive outcomes in many settings, male involvement remains low. The objective of this analysis was to investigate male involvement in maternal and newborn health in Jigawa state, northern Nigeria.

**Methods:**

This qualitative study included 40 event narratives conducted with families who had experienced a maternal or newborn complication or death, in-depth interviews with 10 husbands and four community leaders, and four focus group discussions with community health workers. The interviews focused on understanding illness recognition and care seeking as well as the role of husbands at each stage on the continuum of maternal and newborn health. Data were transcribed, translated to English, and coded and analyzed using Dedoose software and a codebook developed a priori.

**Results:**

This paper reports low levels of knowledge of obstetric and newborn complications among men and limited male involvement during pregnancy, childbirth and the post-partum period in Jigawa state. Men are key decision-makers around the location of the delivery and other decisions linked to maternal and newborn health, and they provide crucial resources including nutritious foods and transportation. However, they generally do not accompany their wives to antenatal visits, are rarely present for deliveries, and do not make decisions about complications arising during delivery and the immediate post-partum period. These gendered roles are deeply ingrained, and men are often ridiculed for stepping outside of them. Additional barriers for male involvement include minimal engagement with health programs and challenges at health facilities including a poor attitude of health providers towards men and accompanying family members.

**Conclusion:**

These findings suggest that male involvement is limited by low knowledge and barriers related to social norms and within health systems. Interventions engaging men in maternal and newborn health must take into account these obstacles while protecting women’s autonomy and avoiding reinforcement of gender inequitable roles and behaviors.

## Plain English summary

Maternal and newborn mortality continue to be major challenges in Nigeria. While greater participation of men in maternal and newborn health has been associated with positive effects, in many settings, male involvement remains low. The main objective of this study was to investigate men’s involvement in pregnancy, childbirth and newborn care in Nigeria. Individual interviews with men and focus group discussions with key respondents were conducted. A thematic approach was used to analyze data.

The findings of the study demonstrate that men have low knowledge about maternal and newborn complications. Men are the main decision-makers around maternal and newborn health, and provide crucial resources and transportation; however, they do not always accompany women to antenatal visits and are rarely present in the delivery room. The key factors which hinder male participation are traditional gender roles, lack of engagement with health programs and poor attitudes of health providers towards men and family members who accompany women.

This study concludes that men have low knowledge and that traditional gender roles and health facility environment are obstacles to male involvement across the spectrum of maternal and newborn health. The study recommends that health programs should increase efforts to engage men and take into account these obstacles. Both men and women should be involved in the design of programs and careful attention should be given to ensure that women’s needs are also met.

## Background

While progress has been made to improve maternal and newborn health over the past decades, maternal mortality continues to be a significant global health challenge [1]. In 2013, there were an estimated 293,000 maternal deaths worldwide, with 99% of these occurring in low income countries [2]. Nigeria is one of the six countries that together contribute more than 50% of the total maternal deaths worldwide [3]. In Nigeria, the most recent national data estimates the mortality ratio (MMR) to be 576 maternal deaths per 100,000 live births (95% CI: 500–652) [4]. Neonatal mortality rates (NMR) at the national level also remain high at 37 deaths per 1000 live births [4]. Both MMR and NMR show wide geographic variation, with the highest rates in the northern regions. A recent study estimated the MMR in Jigawa State in North-Western Nigeria to be 1012 per 100,000 live births, corresponding to a lifetime risk of maternal death of 6.6% [5].

Access to antenatal care (ANC), skilled birth attendance and emergency obstetric care is critical to prevent maternal deaths and severe maternal morbidity [6, 7], yet availability and use of these services in northern Nigeria remain poor [4]. A shortage of skilled birth attendants (SBAs) was partially addressed by the National Primary Health Care Development Agency (NPHCDA) via the Midwives Service Scheme (MSS), entailing deployment of trained midwives to Primary Health Centers (PHCs) to ensure provision of 24-h maternity care [8]. However, this program has not translated to improved skilled birth attendance rates. For example, a recent study found that only 13% of women delivered in a facility with a skilled birth attendant, while 86% gave birth at home with unskilled care [9]. Low maternal health services use in this context is driven by cultural norms, limited support for accessing maternal health services by husbands, and low knowledge of danger signs and available services [10].

Due to their significant role in decision-making in many settings, including in Nigeria, male partner involvement in maternal health has been emphasized as a strategy to improve maternal and newborn health outcomes [11, 12]. For example, male partners may decide if, when and where a woman may access antenatal, delivery and post-partum care, and whether or not to provide financial resources in order to travel to these services [13–15]. However, men who hold decision-making power are often not knowledgeable about pregnancy and childbirth related complications [16, 17]. Previous studies have shown that greater involvement of males during pregnancy has been associated with increased antenatal care visits [18], increased family birth plans [19], and increased delivery with a skilled birth attendant [20].

The Nigerian Federal Ministry of Health (FMOH), in line with the WHO, recommends enhancing male participation in safe motherhood initiatives and reproductive health programs [21]. Despite these recommendations, there is limited research on male involvement during pregnancy and childbirth in Nigeria [22, 23]. One study on spousal participation in labor and delivery in Nigeria found very low levels of male involvement, especially in northern Nigeria, suggesting the need for further research to inform programming [23].

We conducted a qualitative study nested within an ongoing cluster randomized controlled trial (RCT) in Jigawa, northern Nigeria. The primary aim of the qualitative research was to investigate illness recognition, decision-making, and care seeking among families who experienced a maternal death, a reported postpartum hemorrhage (PPH), a neonatal death, or an illness within the first 28 days of life, and the sequences of care seeking actions. This paper presents a secondary analysis of the qualitative data focusing on male involvement in maternal and newborn health.

## Methodology

The qualitative study was conducted between June and November 2015 among families experiencing maternal and neonatal illness and deaths in 24 rural Local Governmental Areas (LGAs) in Jigawa State, Northern Nigeria. The households sampled for the qualitative data collection were drawn from the study population of a large cluster RCT of community-based interventions to reduce maternal mortality in Jigawa State, Northern Nigeria. The RCT was implemented by the Abdul Latif Jameel Poverty Action Lab (J-PAL) and the Planned Parenthood Federation of Nigeria (PPFN) to assess the impact of three interventions: 1) training local women as Community Resource Persons (CoRPs) who provide education and referrals to pregnant women and their families including husbands, 2) the CoRPs program plus distribution of safe birth kits to pregnant women, 3) the CoRPs program plus community dramas to change social norms on maternal health.

The qualitative study included 40 event narratives with families who had experienced a maternal or newborn complication or death, in-depth interviews with 10 husbands and four community leaders, as well as four focus group discussions (FGDs) with CoRPs. The main results of the qualitative study have been presented elsewhere [24]. For this analysis we focus primarily on data from the male and community leader IDIs as well as the FGDs with CoRPs. Data specific to male involvement from the 40 illness narratives are also included in the analysis.

### Study site and background

Jigawa state reported a population of 4.3 million during the 2006 census [25]. The state is divided into 27 LGAs, with 80% of the population living in rural areas [25]. The RCT was conducted in 96 clusters of villages across 24 LGAs, covering an estimated population of 280,000. LGAs were included if they had a PHC that was part of the MSS. The RCT baseline sample consists of women of reproductive age in a 15% subsample of households randomly selected at baseline between December 2011 and May 2012 (*N* = 7069). A RapidSMS surveillance system, where local women were trained in each village to report vital events using text messaging, was implemented to track births and deaths of women and infants. For all births in baseline households, questionnaires were administered within three days after birth, and at 28 days after birth to capture data on the pregnancy, delivery, and post-partum period. For deaths of women of reproductive age, verbal autopsies were conducted to determine cause of death. An endline survey was conducted with the baseline sample between February 2016 and July 2016 (*N* = 6350).

### Study design and sampling method

The qualitative study included data on 40 cases, equally divided between four categories (maternal deaths, reported PPH, neonatal deaths, and neonatal illnesses); maternal and newborn cases were included from both the control and CoRPs arms of the RCT. Potential cases were identified sequentially, using the RapidSMS surveillance system, until the target numbers were reached. Reported PPH, neonatal illness and neonatal death cases were identified via maternal and newborn complication data from the three and 28-day after birth questionnaires, while maternal death cases included those where the death had been verified and a verbal autopsy conducted. Those cases that met the study eligibility criteria via the surveillance data were verified by the field team and then a follow-up visit was conducted for the purpose of qualitative data collection. In some cases, decisions to further pursue eligible cases were based on logistics, cost and geographical considerations. Purposive sampling was then used to select 10 husbands of focal women in maternal or newborn cases as well as four community leaders within the villages where cases were identified who were interested to take part in in-depth interviews. Purposive sampling was also used to identify CoRPs in villages within the RCT’s CoRPs arm who were interested in participating in FGDs.

### Data collection

Fourteen IDIs (10 with husbands, four with community leaders) and four FGDs with CoRPs were conducted between June and November 2015 (See Table 1). Interview guides were developed to explore illness recognition and care-seeking and also included questions on men’s involvement during pregnancy, delivery and post-partum, their involvement in newborn care and their knowledge of danger signs related to maternal and newborn illness. The interview guides were translated to Hausa and piloted extensively. They contained open and closed ended questions on common maternal and newborn complications, perceptions of the severity of these complications as well as appropriate action in response to complications, ideal delivery location, and the specific role of husbands during the spectrum of pregnancy and newborn care, as well as key decision-makers.

**Table 1:** Summary of types and numbers of interviews

Methodology	Target Group	# of Interviews
In-depth Interview	Husband of women who experienced a maternal or newborn complication or death	10
In-depth Interview	Community Leaders	4
Focus Group Discussion	CoRPs – Community health workers who visit pregnant women and their families	4
Illness Narrative	2–3 people present during the following types of cases: 1) maternal deaths, 2) perceived post-partum hemorrhage; 3) neonatal death; 4) neonatal illness	40
TOTAL		58

Data were collected by trained interviewers and note-takers (two male and two female) who conducted interviews in pairs in Hausa. These data collectors were recruited from the study areas specifically for this research and their age and acceptability to the target population were important considerations in the selection process. Interviews were audio recorded and transcribed, and expanded notes were created using notes, memory and the audio. In-depth interviews were on average between 20 and 60 min, while FGDs were between 40 and 90 min.

Supervisors provided ongoing assistance for quality assurance and periodic re-training. Interviewers completed a debriefing template following each interview including additional notes about the data collection process.

### Analysis

Qualitative data were transcribed verbatim by the field team, translated to English and cross-checked for accuracy by Hausa speakers who compared English transcripts to the audio recordings. A codebook was developed a priori and used as a basis for coding the expanded notes. Dedoose software (http://www.dedoose.com/) was used for the coding and analysis of the anonymized data by two researchers (VS, NG). Coding focused on emerging themes related to knowledge of danger signs in pregnancy, during delivery and post-partum as well as in newborns, involvement of men in maternal and newborn health as well as around recognition of illness, decision-making, and patterns of care-seeking. Thematic content analysis was conducted to understand the spectrum of male involvement across the continuum of maternal and newborn health care and whether perspectives varied by type of interviewee (women, men, CoRPs). Data and quotations were summarized in a matrix to allow for comparisons.

### Ethical approval

Verbal and written informed consent was obtained from all respondents. Ethical approval was obtained from the Massachusetts Institute of Technology (MIT) and the Jigawa State Operations Research Advisory Committee (ORAC). The trial is registered at clinicaltrials.gov (NCT01487707, Registered 7 December 2011).

## Results

### Background characteristics

Ten IDIs with husbands, four IDIs with community leaders, four FGDs with 28 female CoRPs and 40 illness narratives with a total of 82 men and women were completed (See Table 2). The husbands were between 23 and 40 years of age, had zero to 15 living children and had completed varying levels of education. Community leaders were older and included men between 30 and 70 years of age with six to eight years of education. The CoRPs were women between 30 and 50 years of age, while the illness narratives included women and men between 18 and 60 years of age. Interviewees from 16 LGAs across Jigawa state participated in the data collection.

**Table 2:** Characteristics of Interviewees

Participant Demographics	Husband IDIs N (%)	Community Leader IDIs N (%)	CoRPs FGDs N (%)	Event Narratives N (%)
# of Interviews	10	4	4	40
# of Participants	10	4	28	82
Sex of Participants	Male	Male	Female	Male & Female
Age Range (Yrs)	23–40	30–70	30–50	18–60
# of Children	0–15	2–15	N/A	0–10
Years of Education	3–23	6–8	N/A	N/A

### Male involvement in maternal care and newborn care

The qualitative data demonstrated limited active engagement of men during maternal and newborn care, and emphasized strict gendered divisions related to both work and physical space. The main themes emerging from the qualitative analysis were related to 1) pregnancy as a dangerous time, 2) men as key decision-makers with limited participation in maternal and newborn health and 3) barriers for men and family members at health facilities. These themes are explored in further detail below.

#### Pregnancy as a dangerous time

Pregnancy and childbirth were consistently reported by male and female respondents as highly dangerous and distressful periods that often lead to fatal consequences. Delivery outcomes were perceived as highly uncertain, and, ultimately, in the hands of God. One male interviewee stated: “Really there is danger in pregnancy, if a woman is pregnant, she never has peace of mind, until she gives birth, and weans the child, it is then that she will know that she is safe.” (Husband 3, Maternal Death). A female respondent explained the fear associated with pregnancy: “Every pregnant woman is in a state of danger, because you are not sure whether you are going to survive. So there is great fear in pregnancy. Your relatives, parents and friends will always remain frightened, and will be praying for your safe delivery.” (Woman, Neonatal Death 4). Another respondent explained about pregnant women: “Only Allah knows if you would survive or not.” (Woman, Neonatal Death 3).

##### Limited knowledge about obstetric and newborn danger signs

Despite unanimously considering pregnancy as dangerous, interviewed men displayed limited knowledge of maternal and newborn danger signs and complications. During pregnancy, danger symptoms reported by male interviewees to affect women included fever, blood shortage, headache, abdominal pain, change in skin color, vomiting and edema (see Table 3). Reported danger signs during delivery included eclampsia, blood shortage, prolonged labor, bleeding, and failure to expel the placenta. Headaches were not considered as abnormal but rather as a sign of impending delivery: “In some cases there is problem of headache. She may complain about headache, so if you are sensible enough you will understand that her delivery is approaching.” (Husband 10, Neonatal Death). Prolonged labor was overwhelmingly seen as a complication that signified the need for care seeking. Dizziness, and the discharge of fluids with a strong odor were also reported danger signs. Two men were unable to mention any symptoms or danger signs during delivery and ascribed this lack of knowledge to the fact that delivery doesn’t involve men. “I don’t know anything about this since I am a male.” (Husband 4, Maternal Death). Only a few male respondents were able to name post-delivery complications. These included bleeding, blood shortage, eclampsia, breast pain, and headache (See Table 3).

**Table 3:**
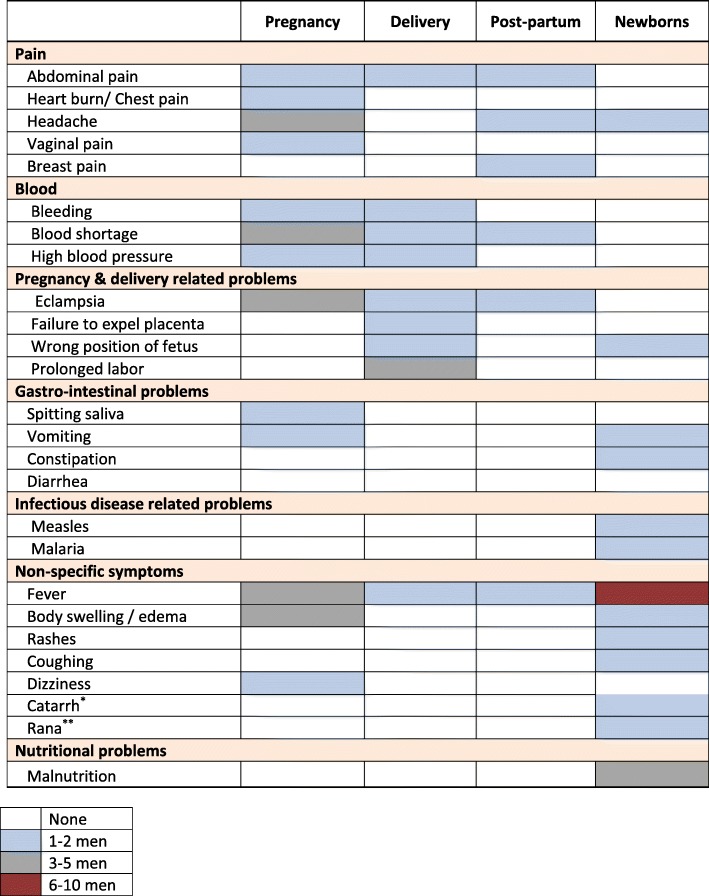
Maternal and newborn symptoms and danger signs identified by male respondents (*N* = 10)

^*^Catarrh refers to excessive discharge or buildup of mucus in the nose or throat

^**^Rana refers to a local illness characterized by a hot body with foamy stool

Symptom recognition by men during pregnancy often relied on visual cues such as alterations in skin color, bleeding, or body swelling, or changes in behavior: “You will see that your wife has changed in the way she will be carrying out her household chores. She will become slow in doing things, and you will also notice that she will not be feeding very well, and from then you can ask her about her condition.” (Husband 10, Neonatal Death). The severity of a symptom was assessed both by its perceived frequency and its link to fatal outcomes “[Fever, convulsions, abdominal pain, body swelling] are the most important because they are the ones to occur the most often. They also kill women.” (Husband 9, Neonatal Illness).

Knowledge of newborn danger signs was also low. The most commonly cited symptoms included fever, cough, excessive crying, and malnutrition caused by insufficient or poor quality breast milk. Respondents also noted that newborn deaths often occur quickly. As with maternal symptoms, newborn symptom recognition relied on visual signs or abnormal behavior such as excessive crying. Men seldom knew the etiology of symptoms and often attributed them to God’s will: “You can find a child who is a year or two years old who has never had a fever… but if God brings it, sometimes you will see a child becoming sick.” (Husband 6, Neonatal Death).

Male respondents reported obtaining maternal and newborn health knowledge from a number of sources including hospitals, friends, personal experience, the radio, and home visits from aid workers. However, they stated that they did not interact or discuss directly with visiting community health workers or CoRPS, and this was confirmed by CoRPS interviews. Some husbands encouraged community health workers to visit their wives but did not partake in activities. Neither men nor women expressed a desire for men to increase knowledge on maternal or newborn health.

##### Mitigating pregnancy risks

Most interviewed men had positive attitudes about their wives attending antenatal care and described it as a means to reduce the likelihood of complications later on: “They will have risks during delivery if they haven’t been to antenatal.” (Husband 2, Maternal Death). However, community health workers describe facing numerous men who are unsupportive of ANC and other types of maternal care. For example, one CoRPs stated: “I visited her [a pregnant woman] several times and asked her to go for antenatal but she didn’t. When I asked her why she said it is her husband that denies her from going and said he will never allow her to…. You see he is not even in town but she is afraid of him and insisted she will never go.” (CoRPS, FGD 4).

While delivery location was often discussed in terms of risks for women, there was no consensus around ideal birth location. Four male respondents expressed positive attitudes towards facility-based delivery because women could be rapidly diagnosed and receive quality care. For example, when asked why he prefers delivery in a facility, one respondent stated: “Because she will have good medical care and any complications will be treated instantly.” (Husband 9, Neonatal Illness). However, one male respondent described his belief that delivery in a health facility is more risky than delivery at home: “If she delivers at home, everything will be fine, you will have peace of mind and you will be proud of this because she has got safe delivery. But if it is at the hospital, you will start thinking about how the delivery is going to happen. Is it going to be a safe one, or will something different happen?” (Husband 7, Maternal Death). Four male respondents reported preferring home-based delivery and visiting the hospital only in case of emergency. Reasons to deliver at home included convenience, a desire for privacy, lack of financial means, and a long tradition of deliveries being performed at home. Several individuals emphasized the importance of ensuring privacy, and of discretion during delivery: “In the village, if a woman delivers inside her room, her secret will not be revealed.” (Husband 8, Neonatal Death).

The most frequently mentioned barriers to facility-based delivery were lack of financial resources and inadequate transportation: “You will see that a person wants to attend the hospital but he hasn’t got the money. Until he finds it or borrows it from someone, it is then that he can be able to go to hospital. That is our problem.” (Husband 4, Maternal Death). Similarly, two respondents mentioned that having access to free and local health facilities staffed by medical personnel were strong incentives to facility-based deliveries. In many communities this remained a problem: “If you visit the hospital during the working hours, you will be treated very well, but as soon as they [health providers] leave, we would then be in a dilemma because there will be no doctor and no birth attendants in the community.” (Community Leader 1).

##### Role of god in coping with poor outcomes

God was stated as a common explanation for complications and outcomes, especially when etiologies were unknown or there were differences in outcomes among women. One respondent explained: “These dangers are God’s will, because we are confused and we can’t understand their causes. You will see that there is a woman that doesn’t have any problems in her pregnancy, and some have problems of vomiting, or spitting saliva very frequently. These are the problems which are occurring, but we have been unable to discover the cause.” (Husband 10, Neonatal Death). Many respondents expressed the belief that reaching medical care is a priority when complications arise. For these respondents, religious faith did not contribute to fatalistic attitudes but rather became an incentive to seek care. As one male respondent described, failure to obtain medical care was seen as defying God’s will: “If you observe that a woman has taken a long time in labor and she didn’t give birth, certainly you know that there is problem, and from there you shouldn’t wait, you just take her to the hospital. And if you said you will wait and see what God is going to do, then know that you have disobeyed God.” (Husband 7, Maternal Death).

In other cases, religious beliefs were found to have impeded care seeking at health facilities. Notably, in a number of cases that ended in a maternal death, the illness or complication was more strongly connected to beliefs around spiritual causes, and families sought various forms of spiritual care including prayers and other rituals, delaying or preventing medical care seeking.

#### Husbands as key decision-makers with limited participation in maternal and newborn health

##### Decision-making around birth location and complications

The hierarchy of decision-makers around birth location appears to follow a specific order. Husbands are unanimously the primary decision-makers with respect to the location of the birth. If he is absent, the husband’s relatives (usually male) assume the responsibility, followed by other designated relatives or neighbors. If these individuals are also absent, then the woman’s parents or other relatives make the decision. In addition, TBAs were reported to participate in decision making at times, and to provide valuable advice, but they were only mentioned by a small number of male respondents.

For certain types of maternal or newborn complications other female relatives made key decisions. For complications arising during or soon after delivery such as post-partum hemorrhage, women who have access to the delivery room are instrumental in the decision to seek care. For most newborn death cases, the newborn’s mother or grandmother made key decisions about care seeking related to the illness. The majority of these deaths occurred soon after delivery, a period in which men have little influence.

##### Husbands as providers of resources

While generally men do not actively participate during labor and delivery, their primary role during this period was described by both women and men as a provider of key resources. During pregnancy, husbands assist their wives by providing the resources needed, including transportation and money, to attend antenatal care or to visit the hospital if complications occur. In some cases, they may be expected to accompany wives to ANC or to send an appropriate representative to accompany them. Husband 4 (Maternal Death) explains that “[Husbands] don’t accompany them, but [they] assist them with transport fees, and also some money for buying food and drugs. So the couples don’t go for antenatal together, unless the delivery comes with complications, it is then that the husband will attend, in case there will be need for something.” Husbands also reported being responsible for providing their wives with nutritious foods that are believed to improve blood and milk supplies before and after birth.

Joint discussions by couples prior to delivery was seen in a positive light, but there was variation in the quantity of discussions and on topics discussed. Most men suggested couples’ discussions during the pregnancy period should center on women informing them when they are not feeling well, but did not describe deeper discussions related to preparedness plans, decisions around delivery location or other decisions. Men interviewed also expressed fear that negative interactions with their pregnant wife could harm the baby: “And you have to be saying good things. If a woman is pregnant and saying bad things... She can talk bad, but don’t show her that you are annoyed, just observe her. Don’t talk back at her even if you are annoyed, just leave her. If God makes it possible that she has a safe delivery, then if you want to separate with her you can but if you put her into a situation, then you have cheated her.” (Husband 2, Maternal Death).

During delivery, the husband’s role is to provide indirect support, as he is not allowed in the delivery room. For example, one woman stated: “Once a woman enters the labor room then there is nothing that the husband can do for her, other than to go home and continue praying for her to have safe delivery. He is only there to provide all the delivery items which are needed. That is all he could do for her, because he would not enter her place (labor room).” (Woman, Maternal Death 10). This norm is so prevalent that even interviewed community health workers did not think that husbands should be present in the delivery room.

However, there were a few exceptions where men have stepped outside of these strongly held traditional norms. One male interviewee explained that “It depends on the husband’s exposure. If you are civilized enough you can go to her. You can even enter the room. But if you are not civilized you will be at the back hiding. You won’t be able to know that she gave birth for almost two days. But for me even if it is in the middle of the night and she is in labor I will go to her.” (Husband 2, Maternal Death). On the other hand, it is not clear whether women themselves would like men to be present in the labor room. In fact, one husband stated that men could be ridiculed for this: “Of course he can [be present in the delivery room], there is nothing wrong about it, but you know among the women, they will start making gossip that his wife has entered a certain situation but he is still present in the place.” (Husband 4, Maternal Death).

Importantly, while the majority of respondents agreed that women should not deliver alone, a few described births with no one present as being a community norm. One respondent stated: “Most women give birth in their rooms without people knowing.” (Husband 6, Neonatal Death). Other respondents reported that other women, usually relatives or neighbors, were acceptable participants in the delivery process. In cases where labor is prolonged, traditional birth attendants (TBAs) were often called to assist.

With respect to newborns, husbands are expected to purchase materials and clothing for the baby, and items for celebration after the birth. One woman summarized her views on the role of the husband: “They should be providing us with nutritious foods like fruits and ‘zogale.’ And then after a woman has given birth, they should provide her with the baby’s clothing, a ram and other items for the naming ceremony.” (Woman, Neonatal Illness 4).

While husbands consistently expressed the strong social norm around male contribution of resources, some women expressed opposing or differing experiences with husbands. For example, one woman stated: “Among we, the Fulani people, the husband doesn’t do anything.” (Woman, PPH 9). Another woman discussed the resourcefulness of some women in coping with their unsupportive partners: “At the hospital, the health workers will explain to you about what you are supposed to be eating. So if you come back, you then inform him. If he likes, he will buy it for you, and if he has no intention of doing it, then you use your own money and buy it for yourself.” (Woman, Neonatal Illness 1). Several female respondents also noted that certain men have a lack of interest or commitment to provide for their wives or are too poor to do so. In these instances, the women were described to have successfully secured resources on their own.

#### Barriers for men and family members at health facilities

Most respondents described numerous challenges at health facilities that impeded care seeking and influenced decision-making. These included the absence of health workers and especially female health providers, lack of drugs or equipment, and health facilities not being open or being too far away. Others described health facilities as not being particularly welcoming for husbands and family members. For example one family member who accompanied a women with complications to the health facility stated: “Immediately on reaching the hospital, she was put on a stretcher and they went away with her… In the village hospital, if you take a patient, they [health workers] will just drive you away from the place and close the door.” (Woman, Maternal Death 2). Several CoRPs also noted that some women and their families were fearful of seeking care at a facility due to the attitudes and behaviors of health providers and the risk of potential mistreatment.

## Discussion

This study reports low levels of knowledge of obstetric and newborn complications among men and limited male involvement during pregnancy, childbirth and the post-partum period in Jigawa State, Nigeria (See Table 4). This pattern may be problematic given that men in this setting provide important resources and make key decisions related to the delivery as well as care seeking for maternal and newborn complications, but do not always have sufficient knowledge to make informed decisions.

**Table 4:** Male involvement along the spectrum of maternal and newborn health care

	Antenatal visits	Delivery	Post natal Visits	Maternal or Newborn Complications
Provides Financial Resources	Yes	Yes	Yes	Yes
Provides Food	Yes	Yes	Yes	N/A
Arranges Transport	Yes	Yes	Yes	Yes
Accompanies wife to facility	Only if complication	Yes, but facility delivery only if complication	No	Yes, if serious
Present in clinic during care	No	No; very strong norms against this	No	Sometimes
Key Decision-maker	Husband	Husband (Delivery Location)	Husband	Husband; Often other women when related to complications in the delivery room or immediate post-partum period
Barriers for greater overall involvement	Gendered norms on roles; men’s lack of knowledge on maternal and newborn health; health facility barriers (health provider attitudes, physical infrastructure of health facilities not conducive to male involvement)			

These findings are consistent with other studies from northern Nigeria and the region [17, 19, 22, 23]. For example, a study in rural Tanzania also reported low levels of knowledge on obstetric danger signs among men, and similar to our findings, knowledge was lowest for danger signs during the post partum periods [17]. Iliyasu et al’s study of 400 men in Kano, northern Nigeria, also found limited knowledge of obstetric complications and low male involvement in maternal health [23]. That study reported that only 13% of men attended at least one ANC visit with their wives and only 18.7% were present during the delivery [23]. It also reported strong cultural and religious factors contributing to low male involvement, and women’s opposition to men being physically present in the labor room. Similarly, our study found strong consensus among men and women that men should not be present in the delivery room, except under exceptional circumstances. One study in Kenya reported higher levels of male knowledge on obstetric danger signs, but this may be explained at least partially by methodological differences in measuring knowledge as well as differences in contextual factors [26].

Our findings highlight the prevalence of strict gendered divisions related to roles and physical space which influence expectations around male involvement including desired knowledge levels. The role of men during pregnancy, childbirth and the post-partum period was described as one of a purveyor of resources, with an emphasis on securing transportation, money and nutritious food. Both men and women expressed similar expectations and socially accepted conventions around male involvement. This is not a surprising finding, and it is consistent with other studies which suggest that socially constructed norms around gender remain a key obstacle to men’s involvement in maternal and newborn health [27–30].

Our findings demonstrate that neither men nor women expect men to have a high level of knowledge related to maternal and newborn health. Rather, other women are seen as the technical resource on these topics. The majority of men do not attempt to acquire additional knowledge and don’t participate in informational visits conducted by community health worker. Men, in general, had positive attitudes towards ANC, but there were differing opinions on the ideal location of delivery, with half preferring home deliveries for their privacy and comfort, and the other half preferring facilities to ensure swift treatment of any potential complications.

A recent systematic review found that increasing male engagement can have positive impacts including improved antenatal care attendance, skilled birth attendance, facility deliveries and post partum care [31]. In Jigawa State, these indicators are amongst the poorest in the country and accordingly are the target of maternal health programming. Our data, however, demonstrate that male involvement has remained low in this setting, and there remain significant barriers to male involvement in maternal as well as newborn health.

For example, the gendered divisions of roles are deeply engrained and men are often ridiculed for stepping outside of traditional gender boundaries [32, 33]. Men, therefore, do not play an active role in delivery unless there is a complication, in which case they can be active decision makers. However, in this setting, it is unclear whether both sexes wish for more active implication of men during the delivery. Caution is needed as several other studies have reported women’s desires to preserve MCH as a women’s domain and to protect their autonomy and safe social spaces [23, 30, 34]. Further research is needed to better understand women’s perspectives in Jigawa on this issue. Additionally, community health programs such as the CORPs program have been unable to systematically engage with men because of these gendered norms and expectations, and there are limited opportunities for men to learn about maternal and newborn health. Finally, similar to several other studies [35–37] health facilities in this area were reported to be particularly unwelcoming to men and family members which also impeded male involvement.

This study has several strengths. First, it includes a sample drawn from 24 distinct LGAs. The study was prospective and involved case-finding using real-time surveillance. These elements increase diversity of the sample and ensure the inclusion of perspectives from a wide geographic area. Data was also collected from female members of the households where men were interviewed which permitted assessment of consensus between males and females in these areas. Limitations include a small sample size of men (*N* = 14). Social desirability bias could have influenced responses, but interviewers were extensively trained in strategies to build trust and rapport with respondents. In addition, given the non-probability sampling approach, external validity may be limited. Finally, men and women who were interviewed had all recently experienced a maternal or newborn complication or death. Viewpoints and perspectives may differ amongst families who have never experienced a complication or death. However, given the high prevalence of complications and deaths in this context, the majority of households would likely have either been directly or indirectly exposed to such events.

Our findings have implications for future health programming, policy and research. Maternal health Interventions developed for northern Nigeria should focus on increasing awareness of danger signs among men and other family members who make key decisions, especially with respect to post-partum and newborn symptoms. This may first require significant effort to change norms and expectations around what men *should* know (i.e., what they are expected to know and desire to know). Maternal health programming should aim to identify and remove barriers for male involvement beyond provision of finance and transportation, but remain sensitive to the needs and desires of women and ensure protection of their autonomy. For example, several studies found negative effects of male involvement including decreased breastfeeding [38] and reduced women’s autonomy [39, 40], as well as reinforced gender inequalities when male involvement programs where implemented in isolation without addressing gender norms [41]. More inclusive approaches that involve both men and women in the design and also that monitor and assess factors such as power dynamics in relationships and unintended consequences are needed.

In addition, programming to remove barriers at health facilities without threatening women’s safe spaces should be developed. For example, a study in Ghana described women’s fears that increased male involvement would turn secure social spaces at health facilities into insecure ones [34]. Other studies have reported that health facilities often do not provide space to accommodate male partners during delivery [42, 43], suggesting the need to consider health facilities and their physical environments. However, interventions must also remain sensitive to the needs of women while promoting alternative constructs of masculinity. As an example, health facilities could potentially provide areas for couples to engage, but also separate spaces for each of the sexes to interact. Finally, further research is needed to better understand how increased male engagement influences maternal and newborn health outcomes, especially related to mortality and morbidity as well as best practices for program implementation in different contexts.

## Conclusion

This study finds low knowledge of obstetric and newborn danger signs among men and limited male involvement across the spectrum of maternal and newborn care in Jigawa State, Nigeria. Men are the key decision-makers with respect to maternal and newborn health, but their main role is to provide resources, food and transportation during pregnancy, delivery and post-partum. Strict gendered divisions related to roles and physical space influence expectations around male involvement including desired knowledge levels in this setting. Health facility barriers including the attitude of health workers are also major hindrances. Efforts to engage men in maternal and newborn health must take into account these obstacles while protecting women’s autonomy. Programs should engage both men and women in the design process and monitor unintended consequences.

## Data Availability

The datasets generated and analyzed during this study are not publicly available since participants did not give consent for the public sharing of their information. However, summaries of the information are available from the corresponding author upon reasonable request. The interview guides for all study participants are also available upon request.

## Abbreviations

ANC: Antenatal Care
CoRPs: Community Resource Persons
FGD: Focus Group Discussion
FMOH: Federal Ministry of Health
IDI: In Depth Interview
J-PAL: Abdul Latif Jameel Poverty Action Lab
LGA: Local Government Area
MIT: Massachusetts Institute of Technology
MMR: Maternal Mortality Ratio
MSS: Midwives Service Scheme
NMR: Neonatal Mortality Rate
ORAC: Jigawa State Operations Research Advisory Committee
PHC: Primary Health Center
PPFN: Planned Parenthood Federation of Nigeria
PPH: Post-Partum Hemorrhage
RCT: Randomized Controlled Trial
SBA: Skilled Birth Attendant
TBA: Traditional Birth Attendant
USAID: United States Agency for International Development
